# Rare complication of bronchoesophageal fistula due to pulmonary mucormycosis after induction chemotherapy for acute myeloid leukemia: a case report

**DOI:** 10.1186/s13256-016-0991-7

**Published:** 2016-07-16

**Authors:** Jun-Hyung Lee, Jin-Soo Hyun, Da-yeong Kang, Hee-Jeong Lee, Sang-Gon Park

**Affiliations:** 1 Department of Internal Medicine, Chosun University Hospital, 365 Pilmun-daero, Dong-gu, Gwangju, 501-717 Republic of Korea; 2 Department of Internal Medicine, Hemato-Oncology, Chosun University Hospital, 365 Pilmun-daero, Dong-gu, Gwangju, 501-717 Republic of Korea

**Keywords:** Mucormycosis, Bronchoesophageal fistula, Neutropenia, Acute myeloid leukemia, Case report

## Abstract

**Background:**

Mucormycosis is a rare and life-threatening invasive fungal infection. Pulmonary mucormycosis commonly occurs in patients with severe neutropenia. Typically, pulmonary mucormycosis causes tissue necrosis resulting from angioinvasion and subsequent thrombosis, so most cases can occur with necrotizing pneumonia and/or hemoptysis. Some complex cases may invade adjacent organs, such as the mediastinum, pericardium, and chest wall. However, to the best our knowledge there is little known regarding bronchoesophageal fistula due to pulmonary mucormycosis after induction chemotherapy for acute myeloid leukemia. We present a case report about this unusual presentation.

**Case presentation:**

A 51-year-old Korean man was diagnosed as having acute myeloid leukemia and received induction chemotherapy. After prolonged severe neutropenia, he complained of coughing with aspiration. Imaging showed a bronchoesophageal fistula with extensive necrotizing pneumonia in the middle and lower lobes of his right lung. Bronchoscopy showed near total tissue necrosis in the middle lobe of his right lung, creating an orifice. A bronchial scope was passed through and was able to be connected with his esophagus; a bronchial wall biopsy was performed. Esophagoscopy revealed a large linear defect of his esophageal wall 30 cm from the incision that may have connected with the bronchus. A bronchial biopsy showed typical hyphae with necrotic tissue, indicating pulmonary mucormycosis. He was given amphotericin B, and a wide excision of lung and esophagus was planned. However, he suddenly died due to massive hemoptysis.

**Conclusion:**

Here we present an extremely rare case of bronchoesophageal fistula with severe necrotizing pneumonia due to pulmonary mucormycosis.

## Background

Chemotherapy-induced immunosuppression has led to an increase in the incidence of fungal infections. Mucormycosis is a rare and life-threatening invasive fungal infection caused by fungi of the Zygomycetes class and Mucorales order. Pulmonary mucormycosis commonly occurs during chemotherapy-induced neutropenia often associated with hematologic malignancy and usually progresses rapidly [1–3].

## Case presentation

A 51-year-old Korean man was diagnosed as having acute myeloid leukemia (acute myelomonocytic leukemia, M4) with normal karyotype (46,XY). An initial complete blood count (CBC) showed a white blood cell (WBC) count of 65.29×10^9^/L, neutrophils 22 %, monocytes 67 %, hemoglobin 8.7 g/dL, and platelet count 18×10^9^/L. He received induction chemotherapy of cytarabine (100 mg/m^2^ body surface area on days 1 to 7) and idarubicin (12 mg/m^2^ body surface area on days 1 to 3).

On day 3 of therapy, when his WBC count reached its nadir, and his absolute neutrophil count was 277/mL, filgrastim was immediately started. He had no signs of infection, including fever, coughing, or diarrhea.

On day 13 of chemotherapy, he developed a neutropenic fever and complained of right pleuritic chest pain with productive coughing and sticky sputum. A chest X-ray showed lobar infiltration of the lower lobe of his right lung. Antibiotics were subsequently changed from prophylactic ciprofloxacin to meropenem and vancomycin.

On day 17 of chemotherapy, his neutropenic fever persisted, and a chest X-ray showed increased infiltration of his right lung field with pleural effusion. Laboratory data were as follows: WBC count of 0.31 (10^3^/mm^3^), neutrophils 51.2 %, lymphocytes 32.2 %, absolute neutrophil count of 150, hemoglobin 7.3 g/dL, platelet count 34 (10^3^/mm^3^), C-reactive protein 27.1 mg/dL (normal range 0 to 0.5), and procalcitonin 1.74 ng/mL (normal range 0 to 0.5). We performed pleural puncture and pleural fluid examinations. Analysis of his right-side pleural fluid indicated an exudative type. His pleural fluid had a WBC count of 670 (42 % neutrophils), total protein 4.75 g/dL, and lactate dehydrogenase (LDH) 1183 IU/L. The results of pleural galactomannan and acid-fast bacilli tests were negative, but the pleural fluid culture grew *Enterococcus faecium* that was resistant to vancomycin.

His antibiotic regimen was switched to meropenem and linezolid, and the prophylactic antifungal agent (oral fluconazole) was changed to caspofungin, an empiric therapy, due to the prolonged neutropenia.

On day 19 of the therapy, his WBC count recovered to an absolute neutrophil count of 3360. A bone marrow biopsy was performed to confirm response to induction chemotherapy, and biopsy results indicated complete remission.

Nevertheless, his fever persisted, a chest X-ray showed no change in lung consolidation, and he complained of a cough that became more severe after swallowing.

**Fig. 1:**
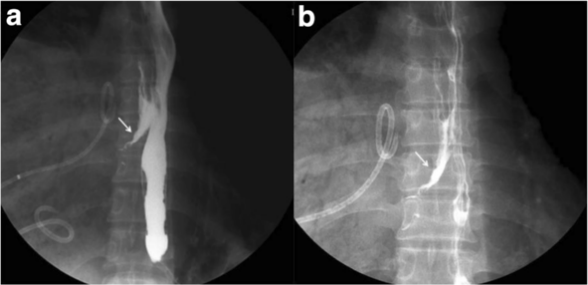
**a**, **b** Barium esophagram shows the fistula between the esophagus and the right bronchus intermedius (*white arrows*)

An esophagram was subsequently performed and revealed an approximately 2 cm filling of a fistula at the mid-esophagus and the right bronchus intermedius (Fig. 1).

**Fig. 2:**
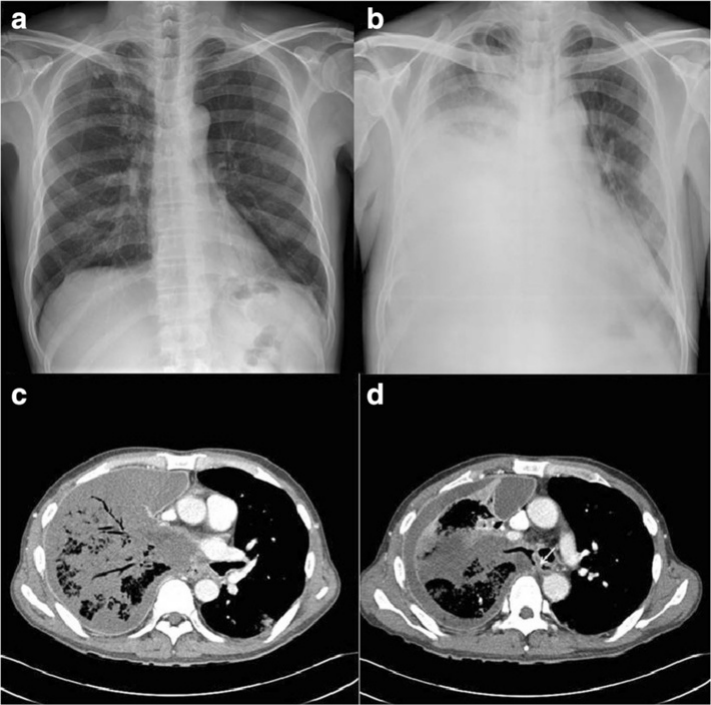
**a** Chest X-ray on the day of admittance. **b** Chest X-ray on day 17 of therapy showing a newly appearing infiltration of right lung with pleural effusion. **c**, **d** Chest computed tomography scan on day 19 of the therapy shows air bronchograms, diffuse consolidation with necrotic low attenuation of right lung (**c**), and bronchoesophageal fistula (*white arrow*; **d**)

Further evaluations with a chest computed tomography (CT) as well as esophagoscopy and bronchoscopy were recommended. Chest CT imaging showed extensive necrotizing pneumonia of his right lung and bilateral pleural effusion predominantly on the right side. In addition, a small, linear, walled air collection was noted just medial to the bronchus intermedius tracking towards his esophagus, a finding suspicious for a bronchoesophageal fistula (Fig. 2).

**Fig. 3:**
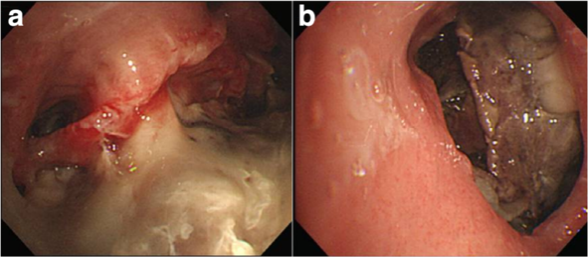
**a** Flexible bronchoscopy showing hyperemic mucosa on right upper bronchus with white exudate and **b** brownish necrotic tissue on the middle lobe of his right lung. Obtained tissue was necrotic

Flexible bronchoscopy showed mucosal hyperemia in his right upper bronchus and brownish extensive tissue necrosis in the middle lobe of his right lung (Fig. 3). Subsequent biopsy and culture were performed.

**Fig. 4:**
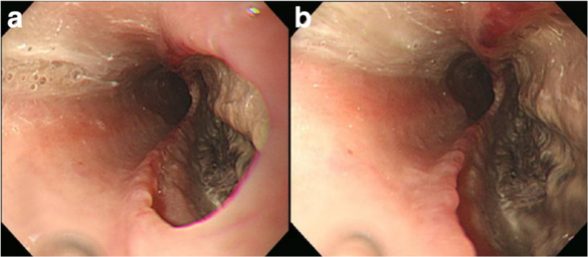
**a** Esophagoscopy showing a large defect of the esophageal wall 30 cm from the upper incision and **b** brownish necrotic tissue

Esophagoscopy revealed a large linear defect of his esophageal wall 30 cm from the upper incision that perhaps connected with the bronchus (Fig. 4).

**Fig. 5:**
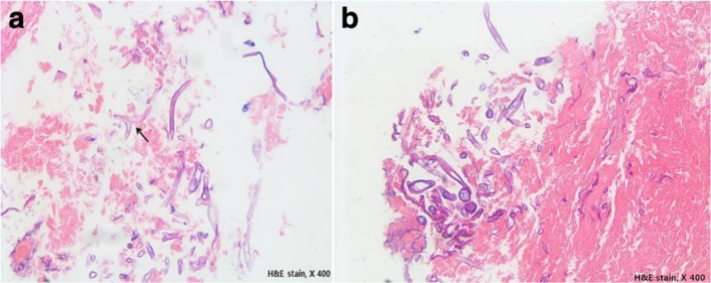
**a**, **b** Bronchoscopic biopsy histology shows many fungal hyphae with necrotic debris. The hyphae appear non-septate and thin-walled. Most hyphae appear broad-branching, with some hyphae appearing to branch at right angles (*black arrow*) upon staining with hematoxylin and eosin (×400)

A bronchoscopic biopsy showed broad and pleomorphic hyphae with necrotic debris. The hyphae appeared non-septate. Branches arose haphazardly, often at right angles. These findings suggested pulmonary mucormycosis (Fig. 5). Lung tissue was cultured; unfortunately, the specimen was inadequate.

The antifungal agent was changed from caspofungin to liposomal amphotericin B (®AmBisome) at a starting dose of 5 mg/kg daily. A wide excision of his lung and perhaps of his esophagus was planned, as continued consolidation chemotherapy was required to treat the acute myeloid leukemia. However, he suddenly died due to massive hemoptysis. A timeline for our patient is given in Table 1.

**Table 1:** Patient’s timeline

Day	Patient’s illness
1	Induction chemotherapy for acute myeloid leukemia (cytarabine + idarubicin) Prophylactic antibiotics (ciprofloxacin)
3	White blood cell count reached it its nadir
13	Neutropenic fever developed Right pleuritic chest pain with productive coughing and sticky sputum Pneumonia developed Changed to meropenem and vancomycin
17	Neutropenic fever persisted Pneumonia aggregation Exudative pleural effusion *Enterococcus faecium* with resistance to vancomycin in pleural effusion Meropenem and linezolid, and caspofungin
19	White blood cell count recovered within normal range Complete remission for acute myeloid leukemia through bone marrow biopsy Developed sudden cough that would become more severe after swallowing Esophagram; thin fistula at the mid-esophagus and the right bronchus intermedius Chest computed tomography imaging; extensive necrotizing pneumonia of the right lung and suspicious for a bronchoesophageal fistula Bronchoscopy; hyperemic mucosa on right upper bronchus and brownish necrotic tissue in the middle lobe of his right lung Esophagoscopy; a large linear defect of the esophageal wall
20	Mucormycosis confirmed Amphotericin B instead of caspofungin Sudden death due to hemoptysis

## Discussion

Mucormycosis is a relatively rare infection compared to species such as *Candida* and *Aspergillus*. The prevalence of mucormycosis is estimated at 1.7 cases per million people per year according to one population-based study and has ranged from 1 to 5 cases per 10,000 according to some autopsy studies [4–6]. Mucormycosis is found in 0 to 8.3 % cases of acute leukemia, and the most common pattern of presentation is pulmonary mucormycosis [1, 7, 8].

Both mononuclear and polymorphonuclear phagocytes of normal hosts kill Mucorales by generating oxidative metabolites and cationic peptides known as defensins. The most important conditions predisposing to mucormycosis include hematologic malignancy, stem cell transplantation, prolonged and severe neutropenia, poorly controlled diabetes mellitus with or without diabetic ketoacidosis, iron overload, major trauma, prolonged corticosteroid use, illicit intravenous drug use, neonatal prematurity, and malnourishment [1–3, 9, 10].

Based on the clinical presentation and involvement, mucormycosis can be divided into the following six major clinical forms: (1) rhinocerebral, (2) pulmonary, (3) cutaneous, (4) gastrointestinal, (5) disseminated, and (6) uncommon rare manifestations, such as endocarditis, osteomyelitis, peritonitis, and renal infection. Among them, pulmonary mucormycosis occurs most often in patients with neutropenia undergoing chemotherapy or stem cell transplantation [11, 12].

The clinical signs and symptoms of pulmonary mucormycosis are nonspecific and cannot be easily distinguished from pulmonary aspergillosis. It usually presents with necrotizing pneumonia, and the symptoms include prolonged high-grade fever and other various findings including cough, chest pain, dyspnea, and hypoxia. In particular, pulmonary mucormycosis results in tissue necrosis due to angioinvasion and subsequent thrombosis; if a major blood vessel is involved, it can lead to hemoptysis. Multiple reports show that in severe cases, it can invade adjacent organs, such as the mediastinum, pericardium, and chest wall [11–15].

The most frequent radiographic findings include infiltration, consolidation, nodules, cavitations, atelectasis, effusion, posterior tracheal band thickening, hilar or mediastinal lymphadenopathy, and even normal findings. However, these findings are indistinguishable from those of pulmonary aspergillosis [16–18]. Therefore, the best mode of diagnosis for pulmonary mucormycosis is bronchoscopic or percutaneous lung biopsy. Ultimately, a confirmatory diagnosis of mucormycosis is based on both: (1) histopathological findings of tissue invasion by hyphae with tissue necrosis resulting from angioinvasion and subsequent thrombosis and 2) cultures isolating pathogens of the order Mucorales [1, 12, 19].

Other than histopathology, there are no biomarkers to identify this disease. Therefore, the high mortality observed in pulmonary mucormycosis may be related to delays in the diagnosis. Another cause of high mortality is rapid progression, extensive involvement, severe underlying conditions, and limited available therapy. The overall mortality rate in pulmonary mucormycosis is high (76 %); it is even higher in severely immunosuppressed patients [1, 12, 20].

Treatment of pulmonary mucormycosis is difficult and often delayed, as described above. Important factors for successful treatment of mucormycosis is rapidity of diagnosis, reversal of the underlying predisposing factor (if possible), appropriate surgical debridement of infected tissue, and appropriate antifungal therapy. Small focal lesions can often be surgically excised before they disseminate or progress to involve critical structures. The only recommended antifungal agents are members of the polyene class, including amphotericin B deoxycholate and its lipid derivatives.

Therefore, the main treatment for pulmonary mucormycosis is immediate surgical resection of the infected tissue, followed by administration of amphotericin B [21–24].

Typically, an esophagopulmonary fistula may occur as a complication of infectious disorders, foreign body ingestion, a complication of intubation with cuff-related tracheal injury, cancer, or congenital anomalies. Pulmonary mucormycosis may invade organs adjacent to the lungs, such as the mediastinum, pericardium, chest wall, or even the esophagus. However, to the best of our knowledge, little is known regarding bronchoesophageal fistula due to pulmonary mucormycosis [25–28].

## Conclusions

Esophagopulmonary fistula is a rare but serious condition. It can occur as a complication of infections but, to the best of our knowledge, there has been no case report of bronchoesophageal fistula due to pulmonary mucormycosis. This is the first case report of such an unusual presentation.

## Abbreviations

CBC, complete blood count; CT, computed tomography; LDH, lactate dehydrogenase; WBC, white blood cell
